# Morphology Matters: Persistent Iatrogenic Aorto-Coronary Dissection Despite Initial Sealing Treated with a Stent-in-Stent Bailout Strategy: A Case Report and Literature Review

**DOI:** 10.3390/reports9030235

**Published:** 2026-07-22

**Authors:** Vincenzo Carfora, Francesco Lanza, Laura Vona, Vittorio Ambrosini

**Affiliations:** 1 Department of Medical Sciences, Division of Cardiology, Sant’Ottone Frangipane Hospital, ASL Avellino, 83031 Ariano Irpino, Italy; vincenzo.carfora@aslavellino.it (V.C.); francescolanza14071995@gmail.com (F.L.); vittorio.ambrosini@aslavellino.it (V.A.); 2 Department of Clinical and Experimental Medicine, University of Foggia, 71121 Foggia, Italy

**Keywords:** iatrogenic aortic dissection, aorto-coronary dissection, percutaneous coronary intervention, coronary stenting, stent thrombosis, stent-in-stent bailout

## Abstract

**Background and Clinical Significance**: Iatrogenic aorto-ostial dissection is a rare but potentially life-threatening complication of percutaneous coronary intervention (PCI), most commonly involving the right coronary artery. Although ostial stenting is generally considered the standard bailout strategy, failure of initial sealing may occur in selected anatomical settings and remains poorly understood. A focused narrative review of the literature was conducted through PubMed/MEDLINE, Scopus and Web of Science to identify reports of PCI-related aorto-coronary dissection with particular attention to dissection morphology, propagation mechanisms, bailout strategies, and outcomes after ostial stenting; **Case Presentation**: A 76-year-old man presented with non-ST-elevation myocardial infarction. Coronary angiography showed severe ostial right coronary artery (RCA) disease and significant left anterior descending artery stenosis. Following drug-eluting stent implantation in the RCA, extensive aorto-ostial dissection with retrograde extension into the sinus of Valsalva occurred. Initial ostial stenting failed to seal the dissection and was complicated by hyperacute stent thrombosis. After successful rewiring of the true lumen, a second overlapping drug-eluting stent was implanted using a stent-in-stent technique, followed by prolonged balloon inflation, achieving complete sealing and stabilization. Serial computed tomography angiography confirmed stability, and staged PCI of the LAD was successfully performed five days later; **Conclusions**: Failure of primary sealing may depend not only on procedural factors but also on dissection morphology. Transverse dissections with wide entry tears may be less effectively sealed by a single ostial stent, whereas overlapping stenting with prolonged balloon inflation may represent a more effective bailout strategy.

## 1. Introduction and Clinical Significance

Iatrogenic aorto-ostial dissection is an uncommon complication of PCI, with an estimated incidence between 0.02% and 0.07% [1].

The right coronary artery (RCA) is most frequently involved, likely due to its anatomical orientation, ostial angulation, and susceptibility to catheter-induced trauma [2].

Iatrogenic aorto-coronary dissection is typically triggered by deep catheter engagement, forceful contrast injection, balloon inflation, or stent deployment in the presence of aorto-ostial disease [1]. Predisposing factors include heavily calcified or eccentric ostial lesions, aggressive catheter manipulation, and the use of guide extension catheters or other devices requiring deep intubation [2]. Although the prognosis has markedly improved with prompt recognition and immediate sealing of the dissection entry site by coronary stenting, extensive aortic extension may still require emergency surgical repair and remains associated with increased morbidity and mortality.

Although many dissections remain localized and benign, retrograde extension into the aortic root can occur, potentially leading to hemodynamic instability, coronary flow compromise, or progression to extensive aortic dissection [3]. Therefore, rapid recognition and immediate management are essential.

We describe a complex case of guide extension catheter-related aorto-ostial dissection during RCA percutaneous coronary intervention (PCI), successfully managed with a stent-in-stent bailout strategy.

### Material and Methods

A focused narrative review of the literature was conducted to identify published reports and reviews concerning iatrogenic aorto-coronary dissection occurring during PCI. A search of the PubMed/MEDLINE, Scopus, and Web of Science databases was performed from database inception through May 2026 using combinations of the following keywords and Boolean operators: (“iatrogenic aortic dissection” OR “aorto-coronary dissection” OR “catheter-induced coronary dissection”) AND (“percutaneous coronary intervention” OR PCI) AND (“ostial dissection” OR “right coronary artery” OR “stent sealing” OR “overlapping stents”). Articles published in English reporting cases of catheter-induced or PCI-related aorto-coronary dissection were considered eligible. Additional references were identified through manual screening of the bibliographies of relevant articles. The search yielded 98 records. After screening titles, abstracts, and full texts for relevance to the scope of this narrative review, 17 publications were included. Attention was given to procedural mechanisms, anatomical predictors, extension patterns, bailout strategies, and outcomes after ostial stenting.

Reports describing failure of initial stent sealing, propagation despite PCI treatment, use of overlapping stents, or prolonged balloon inflation were specifically reviewed to explore possible morphology-dependent mechanisms underlying persistent dissection propagation. Given the rarity of the condition and the predominance of case reports and small case series in the literature, a qualitative narrative synthesis was performed rather than a formal systematic review or meta-analysis.

## 2. Case Presentation

A 76-year-old man with cardiovascular risk factors including hypertension, dyslipidemia, and a history of smoking (former smoker) presented to the emergency department with oppressive chest pain and dyspnea.

On admission, the patient was hemodynamically stable (blood pressure 150/90 mmHg, heart rate 85 bpm, oxygen saturation 96% on room air). Electrocardiogram showed inferolateral ST-segment depression. High-sensitivity troponin was elevated (5736 ng/L; normal < 11 ng/L).

Transthoracic echocardiography demonstrated mildly reduced left ventricular ejection fraction (47%) with inferior wall hypokinesia and no evidence of aortic dilation or pre-existing dissection.

**Figure 1:**
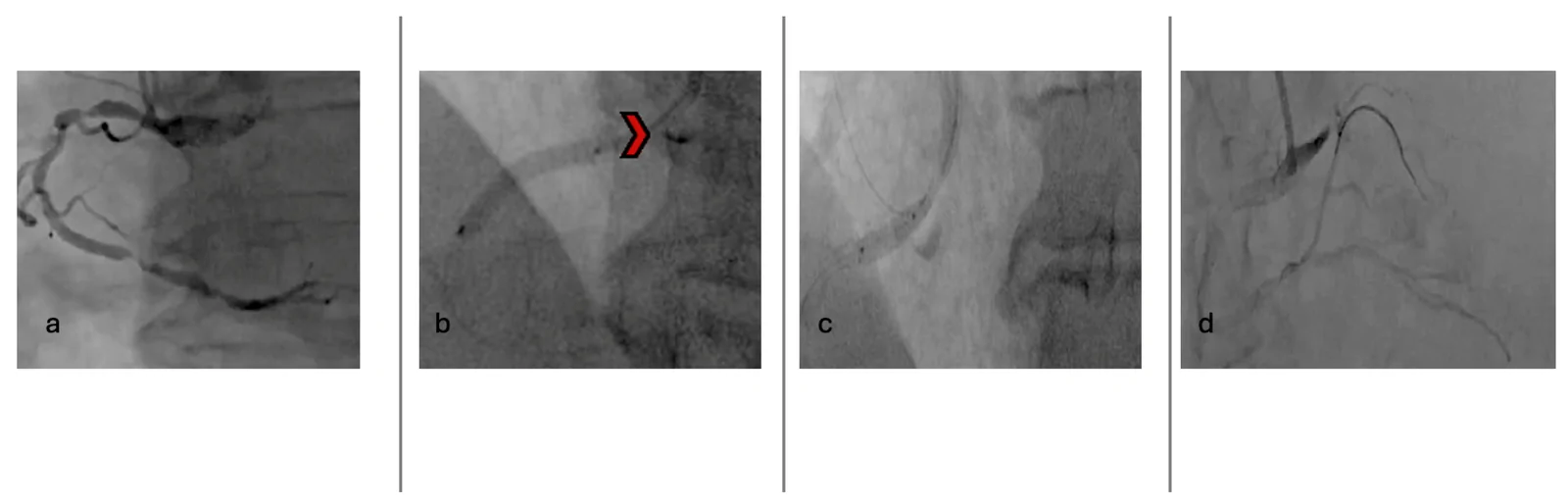
Right coronary artery angiography (**a**). Stent implantation in the mid–proximal segment delivered via guide catheter extension. The red arrow highlights early contrast stagnation within the sinus of Valsalva (**b**). Attempt to seal the dissection entry point with an ostial stent using the floating wire technique (**c**). Progression of the aortic dissection (**d**).

Urgent coronary angiography revealed two-vessel coronary artery disease, consisting of a subocclusive lesion of the RCA (Figure 1a) and a significant stenosis of the LAD.

Given the clinical presentation, PCI of the RCA was undertaken as the culprit lesion.

### 2.1. Procedural Details and Complication

PCI was performed through a left radial artery approach using a 6 Fr JR4 guiding catheter. To improve guide support and facilitate device delivery across the ostial lesion, a 5.5 Fr Boosting guide extension catheter was advanced into the proximal RCA. Anticoagulation consisted of 6000 IU of unfractionated heparin administered at the beginning of the procedure. Fifteen minutes after heparin administration, the activated clotting time (ACT) was 270 s. The right coronary artery was crossed with a 0.014-inch workhorse guidewire, and lesion preparation was performed using a 3.5 × 20 mm semi-compliant balloon inflated to 12 atm. A 3.5 × 28 mm sirolimus-eluting stent was then deployed in the mid-proximal RCA at 12 atm. Angiographic control unexpectedly revealed an aorto-ostial dissection with retrograde extension into the sinus of Valsalva (Figure 1b).

**Figure 2:**
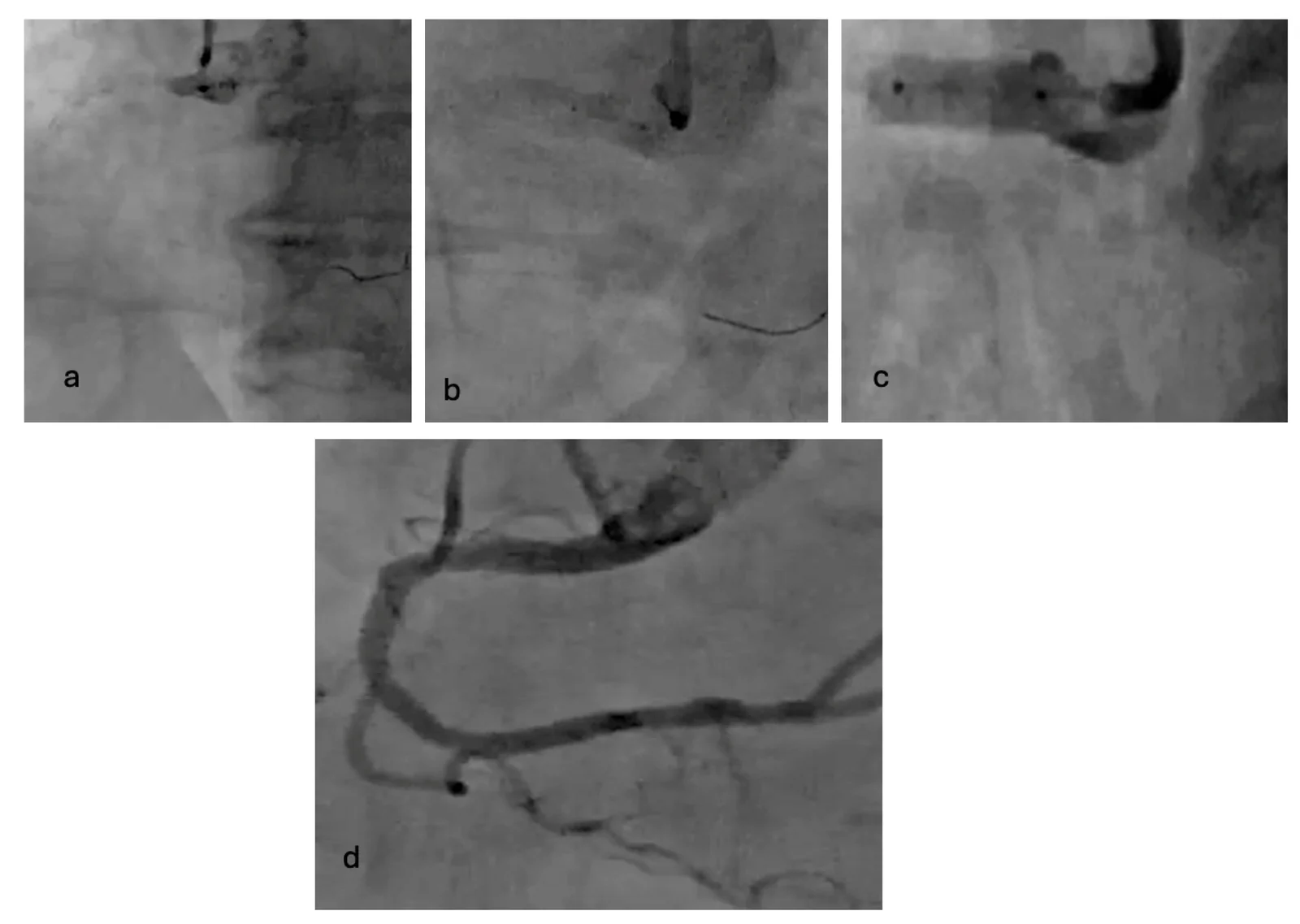
Hyperacute in-stent thrombosis (**a**). Recanalization was achieved, followed by ostial stent-in-stent implantation (**b**) Prolonged inflation of a 5.0 mm balloon for 60 s, resulting in stabilization of the aortic dissection (**c**). Persistent contrast staining in the sinus of Valsalva was observed, without further progression (**d**).

An immediate attempt to seal the dissection entry point with a 4.0 × 13 mm polymer-free amphilimus-eluting stent was performed using a floating wire technique, maintaining a second guidewire within the aortic root to facilitate accurate ostial positioning while preserving access to the true lumen (Figure 1c). However, this resulted in further propagation of the dissection (Figure 1d) and was complicated by hyperacute stent thrombosis, leading to abrupt flow compromise (Figure 2a). Immediately after angiographic evidence of hyperacute stent thrombosis, repeat ACT measurement was 233 s. An additional 3000 IU of unfractionated heparin was administered, and repeat ACT measured 15 min later was 264 s.

At this stage, the procedure became critical. Careful re-wiring of the true lumen was successfully achieved, restoring access to the distal vessel. A second ostial drug-eluting stent, consisting of a 4.0 × 13 mm polymer-free amphilimus-eluting stent, was implanted within the previously deployed ostial stent using a stent-in-stent technique at 12 atm (Figure 2b), followed by post-dilatation with a 4.5 × 15 mm semi-compliant balloon inflated to 16 atm. Finally, prolonged inflation of a 5.0 × 10 mm non-compliant balloon was performed at 14 atm for 60 s, using a prolonged balloon inflation strategy similar to that commonly adopted for the treatment of coronary perforations (Figure 2c). This maneuver resulted in complete sealing of the dissection and restoration of normal TIMI 3 coronary flow (Figure 2d). Intravascular imaging was not performed because the patient’s clinical condition required immediate bailout treatment.

### 2.2. In-Hospital Course and Follow-Up

**Figure 3:**
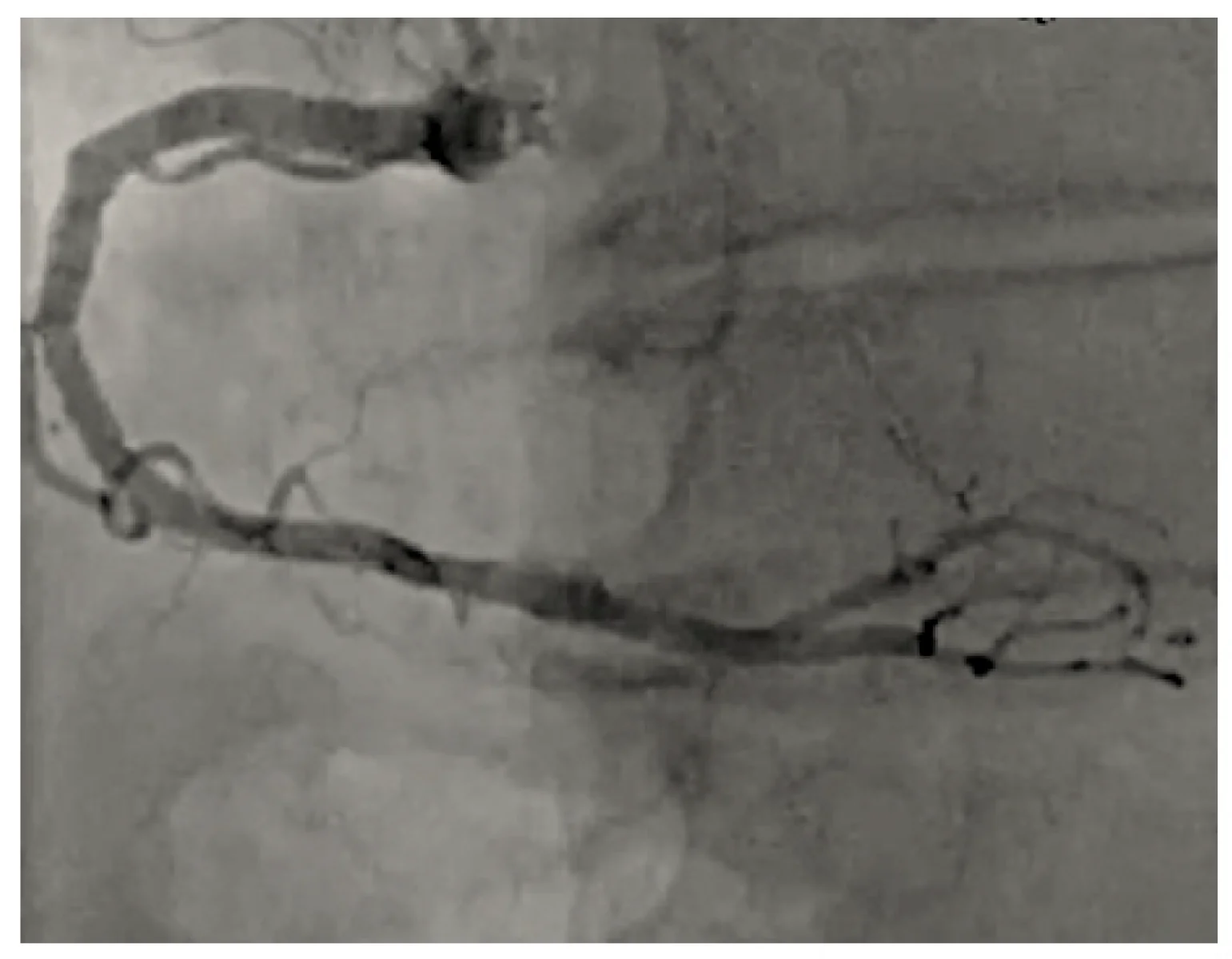
Five-day angiographic control showing an optimal final result, with no residual contrast staining in the sinus of Valsalva.

The patient was transferred to the coronary care unit for close monitoring. Serial transthoracic echocardiography showed no worsening of ventricular function or pericardial complications. Computed tomography angiography performed at 1 h and 48 h confirmed stability of the aorto-ostial dissection, with no progression into the ascending aorta (Figure 3).

Five days later, a staged PCI of the LAD was successfully performed without complications. The patient was discharged on day 7 on dual antiplatelet therapy (aspirin and ticagrelor).

**Figure 4:**
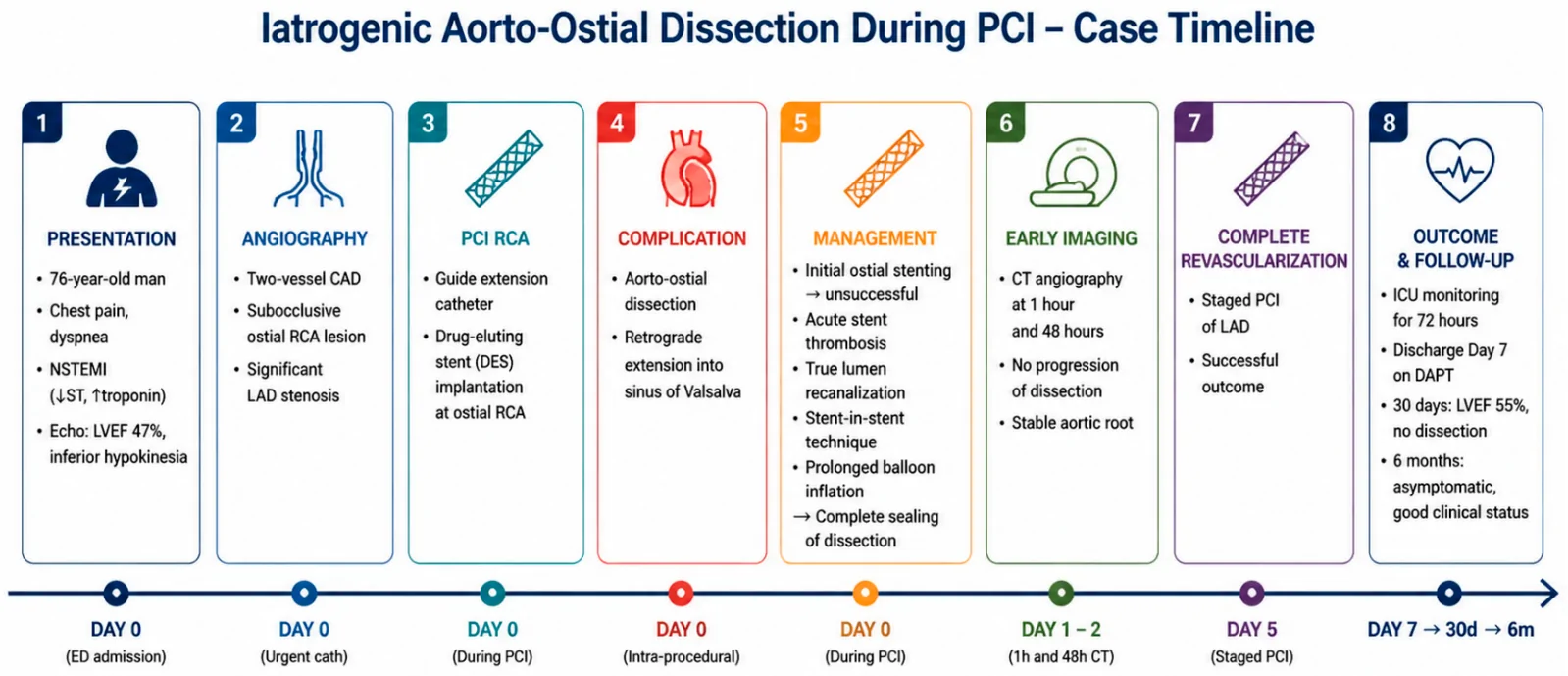
Timeline of the case showing presentation with NSTEMI, coronary angiography findings, PCI of the RCA complicated by aorto-ostial dissection, percutaneous management with stent-in-stent technique, and clinical follow-up. The figure was generated using Google Gemini (AI-assisted illustration) https://gemini.google.com/app?hl=it (accessed on 14 May 2026).

At 30-day follow-up, echocardiography demonstrated normalization of left ventricular function (ejection fraction 55%) and absence of wall motion abnormalities. At 6 months, the patient remained asymptomatic and repeat transthoracic echocardiography confirmed preserved left ventricular function with no new abnormalities. Follow-up computed tomography was recommended but declined by the patient; therefore, no additional CT assessment of the aorta was available. Figure 4 summarizes the procedural timeline and management of the iatrogenic aorto-ostial dissection.

## 3. Discussion

Iatrogenic aorto-ostial dissection is an uncommon yet potentially catastrophic complication of percutaneous coronary intervention (PCI), most frequently involving the ostium of the RCA [3]. The greater susceptibility of the right coronary artery to iatrogenic aorto-coronary dissection during PCI appears to mainly reflect anatomical and microstructural differences in the coronary ostium and vessel wall, with heterogeneity in the arrangement of collagen and elastin, as well as a higher frequency of RCA involvement in aorto-coronary dissections reported in the literature [4].

The underlying pathophysiology is multifactorial, reflecting the interplay between procedural mechanics and the intrinsic vulnerability of the aorto-ostial segment [5]. In a systematic review of published cases of iatrogenic aorto-coronary dissection during PCI, the right coronary artery was the most frequently involved vessel (74.8%), and the reported cases were predominantly male (81.3%) [6].

Rapid recognition of an aorto-coronary dissection in the catheterization laboratory is of critical importance, as timely diagnosis and immediate management may prevent further extension of the dissection and catastrophic clinical deterioration. The extent of aortic involvement is commonly described according to the Dunning classification: type I, confined to the coronary cusp; type II, extending into the ascending aorta by <40 mm; and type III, extending into the ascending aorta by ≥40 mm [7].

Although the classification can be established immediately during the procedure, the most accurate and precise modalities are computed tomography (CT) or, alternatively, transesophageal echocardiography (TEE) [8].

In general, type I and II dissections may be managed conservatively in carefully selected hemodynamically stable patients or treated by ostial stenting to seal the entry tear and prevent further propagation. Type III dissections have traditionally been considered an indication for urgent surgical repair; however, selected cases reported in the literature have also been successfully treated with coronary ostial stenting, particularly when prompt sealing of the dissection entry was achieved and the patient remained stable [9,10].

In the largest published systematic review of iatrogenic aortocoronary dissections during right coronary artery procedures, ostial stenting was performed in 62% of cases, conservative management in 18%, and surgery in 28%. Notably, type III dissections accounted for 54% of cases, with an in-hospital mortality of 6.5% in this subgroup [9].

The need for surgical repair in these patients appears to be lower than in spontaneous aortic dissection, likely because the underlying substrate is fundamentally different. In iatrogenic aortocoronary dissection, the lesion is typically focal, procedure-related, and often recognized immediately, which allows rapid sealing of the entry tear and limits further propagation. By contrast, spontaneous aortic dissection usually reflects a more diffuse aortic wall pathology and therefore more frequently requires surgical intervention [11].

Several mechanisms contribute to the development of this complication. Direct mechanical trauma from guiding catheters—particularly when deeply engaged—represents one of the primary triggers [5]. Balloon inflation at or near the ostium can further increase wall stress, especially in vessels with reduced elasticity or underlying atherosclerotic disease [12]. In addition, forceful contrast injection in a suboptimal catheter position may generate localized high-pressure gradients capable of disrupting the intimal layer. Device manipulation, including wires, balloons, and stents, may also promote the progression of small intimal injuries into more extensive dissections [13].

In this case, the use of a guide extension catheter appears to have played a central role in the onset of the dissection. Deep intubation, while often necessary to enhance support and facilitate device delivery, reduces antegrade blood flow and limits pressure dissipation [14]. When combined with contrast injection, this setup can generate a high-pressure jet within a confined lumen, significantly increasing shear stress on the vessel wall. This effect is further amplified by reduced backflow during deep engagement. In addition, the biomechanical changes induced by initial stent deployment—such as localized stiffening of the vessel—may predispose the artery to further injury and facilitate retrograde propagation of the dissection toward the aortic root. In this setting, the stent itself may inadvertently act as a pathway for proximal extension of the dissection.

The initial attempt at ostial stenting was unsuccessful, likely due to incomplete sealing of the dissection entry point, inaccurate identification of the origin of the dissection, or continued propagation during the procedure, despite the use of the floating wire technique [15].

Hyperacute stent thrombosis represents a critical complication that may occur after complex bailout interventions. In this case, several factors may have contributed, including extensive manipulation of the ostial segment, multiple stent layers, acute dissection-related flow disturbance, and possible delayed endothelial sealing. Although ACT values were monitored and additional unfractionated heparin was administered after thrombosis onset, the occurrence of thrombosis highlights the complex thrombotic milieu associated with emergency ostial stent implantation [16].

**Figure 5:**
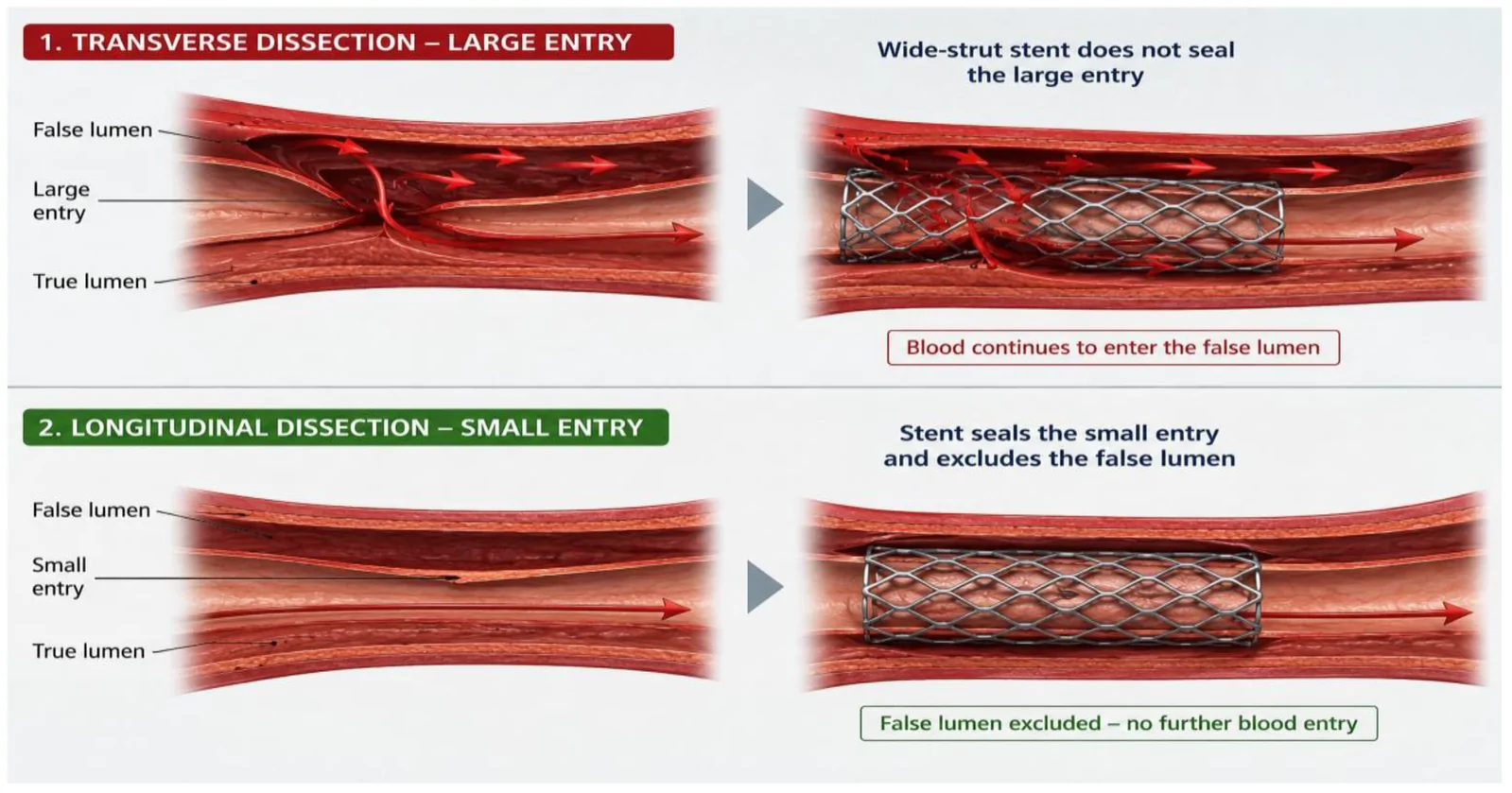
Schematic representation of the proposed dissection morphology. This illustration is conceptual and is intended to depict a possible mechanism underlying persistent false lumen communication; it does not represent direct anatomical imaging. The figure was generated with the assistance of ChatGPT (GPT-5.5, OpenAI) and subsequently reviewed and adapted by the authors (accessed on 16 May 2026).

The failure to achieve complete sealing of the aorto-coronary dissection with the initial ostial stent implantation, together with the occurrence of immediate stent thrombosis, raises the possibility that the dissection was characterized by a transverse tear orientation with a relatively wide entry site. In this setting, the stent-in-stent technique may have improved sealing by increasing radial support and metal coverage at the entry site, thereby providing more effective vessel scaffolding and stabilization, although this mechanism remains hypothesis-generating and the effectiveness of this approach has not been consistently demonstrated in the literature [17] (Figure 5).

**Figure 6:**
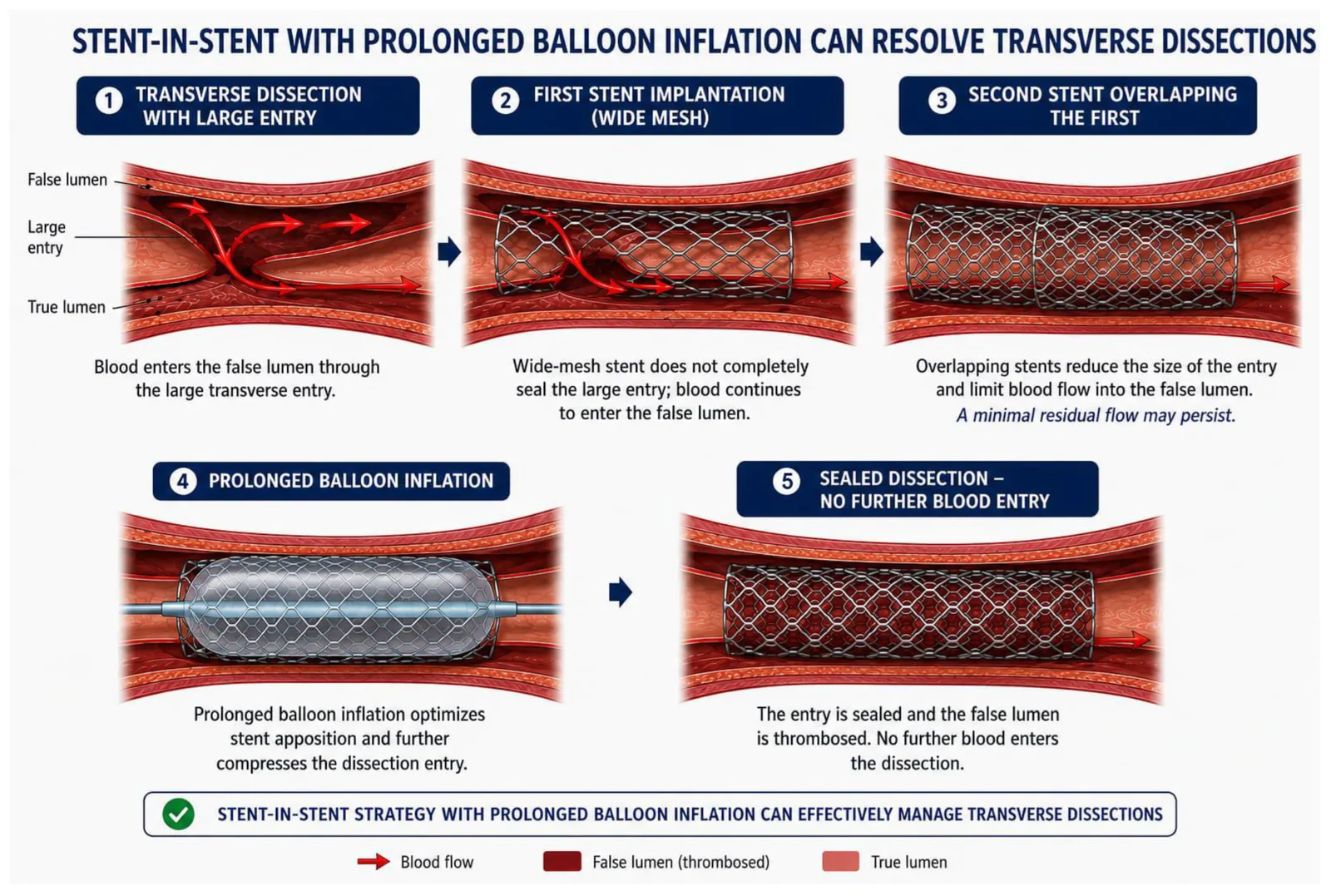
Schematic representation of the proposed mechanism of entry-site sealing using a stent-in-stent strategy combined with prolonged balloon inflation. The figure is conceptual and does not represent direct anatomical evidence. The figure was generated with the assistance of ChatGPT (GPT-5.5, OpenAI) and subsequently reviewed and adapted by the authors (accessed on 16 May 2026).

In this setting, a single stent may not always provide adequate sealing of a large or persistent entry site, particularly when continuous communication with the false lumen remains [18,19]. In our case, the stent-in-stent technique was considered to provide additional metal coverage at the level of the entry tear through strut overlap, while prolonged balloon inflation was used with the aim of promoting further sealing of the dissection plane (Figure 6). This approach should be considered hypothesis-generating, as the optimal management strategy for different dissection morphologies remains undefined.

Although covered stents may provide immediate sealing of coronary dissections [20], their use may be limited by concerns regarding thrombogenicity, reduced flexibility, and technical challenges, particularly in ostial locations [21]. In the present case, the stent-in-stent approach followed by prolonged balloon inflation was selected as a bailout strategy, allowing additional scaffolding and controlled treatment of the dissection while avoiding the potential limitations associated with covered stent implantation.

Intravascular imaging techniques such as intravascular ultrasound (IVUS) could have provided additional insights into the extent and morphology of the dissection, as well as guidance for optimal stent deployment [22]. However, in the acute setting, the priority remains rapid restoration of coronary flow. We elected not to prolong the procedure, particularly in light of the hyperacute stent thrombosis.

Therefore, although the proposed dissection morphology may have influenced the treatment response, this remains a hypothesis and should not be considered the sole explanation for the procedural outcome. Other procedural and technical factors may also have contributed, including the complexity of the ostial RCA anatomy, possible incomplete coverage of the dissection entry site, mechanical interaction between devices, and persistent blood flow into the false lumen.

Overall, although iatrogenic aorto-coronary dissections have been previously described, this case provides a unique procedural perspective by documenting failure of initial ostial sealing, the occurrence of hyperacute stent thrombosis, and successful bailout management with a stent-in-stent strategy combined with prolonged balloon inflation. It underscores the complexity of managing this rare complication and highlights the importance of technical awareness, careful device handling, prompt recognition, and procedural adaptability. Furthermore, it suggests that dissection morphology may represent one of several factors influencing procedural decision-making, although this hypothesis requires confirmation in future studies.

## 4. Conclusions

Aorto-ostial dissection is a rare but potentially catastrophic complication of PCI, particularly when guide extension catheters are used. Although ostial stenting is generally considered the first-line bailout strategy to seal the entry tear and prevent retrograde propagation, this case highlights that initial sealing may fail and may be complicated by hyperacute stent thrombosis. Successful management was achieved with a second overlapping stent using a stent-in-stent strategy combined with prolonged balloon inflation, resulting in complete sealing of the dissection and restoration of coronary flow. This case emphasizes the importance of prompt recognition, careful device handling, and procedural adaptability in the management of complex iatrogenic aorto-coronary dissections. Further studies are required to better define the optimal treatment strategies for these challenging scenarios.

## Data Availability

The original contributions presented in the study are included in the article. Further inquiries can be directed to the corresponding author.

## Abbreviations

The following abbreviations are used in this manuscript:

PCI	Percutaneous coronary intervention
RCA	Right coronary artery
LAD	Left anterior descending artery
NSTEMI	non–ST-elevation myocardial infarction
